# Systematic identification of rare disease patients in electronic health records enables evaluation of clinical outcomes

**DOI:** 10.1038/s41598-026-43020-x

**Published:** 2026-04-18

**Authors:** Arjun S. Yadaw, Eric Sid, Hythem Sidky, Chenjie Zeng, Qian Zhu, Ewy A. Mathé

**Affiliations:** 1 Division of Preclinical Innovation, National Center for Advancing Translational Sciences (NCATS), NIH, 9800 Medical Center DR-BLDG B, RM 366, Rockville, MD 20850 USA; 2 Division of Rare Diseases Research Innovation, Sciences (NCATS), National Center for Advancing Translational, National Institutes of Health, Bethesda, MD 20892 USA; 3 Precision Health Informatics Section, National Human Genome Research Institute, National Institutes of Health, Bethesda, MD 20814 USA; 4 Schools of Medicine, Public Health, and Nursing, Johns Hopkins University, Baltimore, MD 21287 USA

**Keywords:** Rare disease, Rare diseases mappings, Retrospective analysis, ICD-10, SNOMED-CT, N3C, COVID-19 outcomes, Cardiology, Computational biology and bioinformatics, Diseases, Health care, Medical research

## Abstract

Identifying rare disease (RD) patients in electronic health records (EHRs) is difficult, as most of the over 10,000 RDs are not adequately captured by standard coding systems. To address this, we developed a semi-automated workflow to map RDs to SNOMED-CT and ICD-10 codes, enabling improved RD identification across EHR systems. The optimized workflow yielded 88.4% true RD codes in a subset of 1,715 manually curated diseases. Using this workflow and starting with 12,003 GARD IDs mapped to ORPHANET, we obtained 12,081 SNOMED-CT and 357 ICD-10 codes representing 6,342 RDs, organized into 30 ORPHANET linearization classes. We applied these codes to the National COVID Cohort Collaborative (N3C) dataset of over 21 million patients. Among these patients, 8.46 million were identified as COVID-19 positive, of which 4.8 million were used in analyses. Among these, 316,836 (6.55%) had a preexisting RD. Logistic regression, adjusted for age and BMI, revealed that most RD classes were significantly associated with increased odds of severe COVID-19 outcomes. Notably high odds of mortality were observed for rare cardiac (OR = 4.07) and otorhinolaryngologic diseases (OR = 4.00). Hospitalization risk was also elevated across all RD classes, with the highest odds seen in otorhinolaryngologic (OR = 4.31) and endocrine diseases (OR = 3.38). This approach enables scalable RD patient identification in EHRs and highlights the need for tailored healthcare strategies to improve outcomes in RD populations.

**Supplementary Information:**

The online version contains supplementary material available at 10.1038/s41598-026-43020-x.

## Introduction

Currently, 25 to 30 million individuals in the United States (US) and over 300 million people across the world are diagnosed with a rare disease (RD)^1–3^. This means that 3.5–5.9% of the world’s population is affected by RDs^3^. Collectively, RDs represent a significant public health burden with more than 10,000 identified RDs^1^. Among them, only 5% have FDA approved therapeutic options^1^. Indeed, developing interventions for a single RD is very costly and timely, and drug development costs are recouped by a limited number of patients, resulting in markedly higher costs of orphan drug treatments than non-orphan drugs, particularly in the US^4^. Notably, our ability to identify and evaluate this patient population as a whole or as groups, rather than one disease at a time, provides opportunities for finding shared interventions. It thus becomes possible to leverage shared information about diseases, including similarities in genetic or other molecular profiling, mechanisms or action, symptoms, to group them for drug repurposing efforts or implementation of basket trials^5^. In this work, we focus on identifying RD patients in electronic health records (EHRs), as these provide a rich source of information for translational research efforts that aim to understand disease patterns, find new potential treatments and guide implementation of clinical trials^6–8^.

Identification of RD patients in EHRs remains challenging due to the heterogeneity of the coding systems used across hospital systems and due to the lack of a comprehensive list of RD-specific codes^9^. In the US, most hospital systems rely on the Health Insurance Portability and Accountability Act (HIPAA)-compliant standardized ICD-10-CM codes to report disease diagnoses^10^. Importantly, these systems are primarily put in place for billing and business assessments^11^. An independent and complementary standard nomenclature established in 1999, Systematized Nomenclature of Medicine—Clinical Terms (SNOMED-CT), provides a means for comparing clinical healthcare information across different institutions and can be mapped to the ICD-10^12^. In the United States, the ICD-10 and SNOMED-CT codes are heavily used in EHR systems to capture diagnosis-related information and standardized complementary information on diseases, respectively. Another highly utilized and relevant standard is the Observational Medical Outcomes Partnership (OMOP) Common Data Model (CDM)^13^ which produces standardized vocabularies that group differing medical terms across disparate clinical systems into a standardized concept. Using such standardized concepts enables the integration and cross-evaluation of data across different systems that are each collecting data using their own standards and criteria.

In parallel, large national and international initiatives are providing detailed, complementary information on RDs to further enhance data cross-linking and definitions of diseases. In the United States, the GARD (Genetic and RDs Information Center) provides access to up-to-date research, resources and diagnostic guidance to RD patients^1^. The Monarch Initiative^14^, an international consortium aggregating and harmonizing resources on human genes and diseases, model organisms, genomic data, and expression/pathway information, produces MONDO (Monarch Merged Disease Ontology)^15^. MONDO provides an ontology for connecting diseases across databases and resources. ORPHANET^16^, an international effort supported by the European Commission, provides information on RDs in many different languages as well as orphan drug and RD inventories. Importantly, these resources cross-reference each other to promote interoperability and cross-sharing of information, which enables the RD code generation introduced in this study^1,12,16,17^.

There are two broad approaches to identifying RD patients in EHRs, those based on knowledge mining of EHR records and those based on the use of standardized codes. One example of the former includes the use of EHR records to learn text embeddings, which are in turn used to predict the likelihood of patients having an RD or lipodystrophy in this case^18^. In another study, acute hepatic porphyria patients were predicted via support vector machines with information on diagnosis, medications, procedures and clinical notes as input^19^. AI-based digital health assistants have also been applied to identify RDs in EHR records, largely using symptom data^20,21^. In contrast, other studies rely heavily on standardized codes. For example, one study performed on Asian healthcare system data utilized a value set of SNOMED-CT codes with demographic and symptom filters to evaluate patients with Fabry disease and familial hypercholesterolemia (rare genetic disease)^22^. Another recent study developed algorithms to identify patients with Gaucher disease (GD) in an EHR system using ICD-10 codes and standardized laboratory results and symptoms^23^. Globally, these studies aim to ameliorate the process of identifying RD patients in EHR records to help shorten the diagnostic odyssey length.

Nonetheless, it is worth noting that a recent study comparing the performance of LLMs in diagnosing diseases demonstrated that more traditional methods still outperforming LLMs^24^. Furthermore, predictions via knowledge mining are typically focused on single diseases and it is currently not feasible to develop such an algorithm for all RDs. Furthermore, while the use of standardized codes is applicable to a broader set of diseases, a comprehensive starting list of disease codes is needed. We note that there is currently no gold standard covering more than 10 K existing RDs that are EHR system type agnostic. To the best of our knowledge, no algorithm to date has aimed to identify all US-defined RDs in EHRs.

**Fig. 1:**
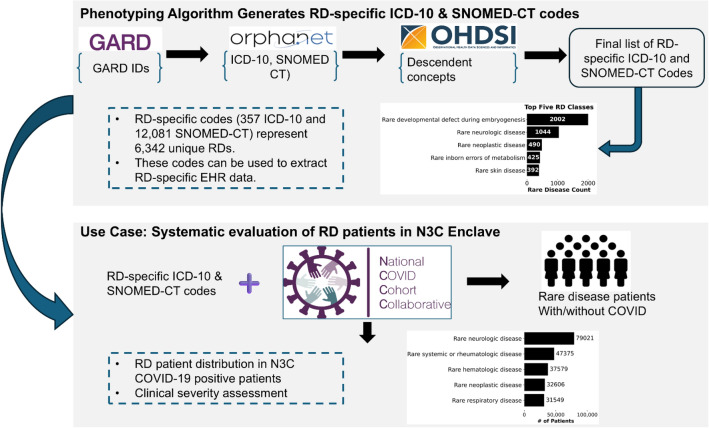
Study design. In this study, we developed a phenotyping algorithm that leverages GARD, ORPHANET, and OHDSI to produce RD-specific ICD-10 and SNOMED-CT codes. These codes are specific to individual diseases that devoid group of disorders and phenotypes. We demonstrate the utility of these codes through a clinical assessment of RD patients in the N3C Enclave. Specifically, we systematically identified RD patients in the enclave and evaluated distributions of RD patients by their ORPHANET class, as well as risk of COVID-19 mortality.

In this study, we aimed to develop an automated process for generating RD-specific ICD-10 and SNOMED-CT codes for systematic identification of RD patients in EHRs. We recognize the continued impact of COVID-19 with more than 7.09 million deaths worldwide, as of February 23^rd^, 2025^25^ and thus exemplify the utility of this list of RD-specific codes to assess the potential impact of COVID-19 on RD patients. We mapped these RD-specific codes to OMOP concepts to identify RD patients in the National COVID Cohort Collaborative (N3C)^26,27^. Patients with RD prior to COVID-19 diagnosis were further classified into ORPHANET linearization groups to evaluate their risk of COVID-19 severity outcomes using logistic regression models adjusted for age and BMI. Overall, this study produces a list of RD-specific SNOMED-CT and ICD-10 codes that can be used to identify patients in EHR, as we exemplify in evaluating COVID-19 clinical outcomes within the RD population in N3C (Fig. 1).

## Methods

### Development of the RD phenotyping algorithm

As a starting point, we compiled a curated RD list of 12,003 comprising GARD IDs, where each represents a unique RD. GARD maintains a comprehensive registry of RDs, with particular emphasis on conditions meeting the United States regulatory definitions of rare disease status. This registry incorporates external citations to authoritative sources regarding disease etiology, diagnostic criteria, and therapeutic management protocols^1^. These GARD IDs were mapped to ORPHANET concepts (July 2023 version)^16^ which have been mapped to SNOMED-CT and the ICD-10.

These retrieved SNOMED-CT and ICD-10 mappings then underwent a series of filtration steps to produce the final list of RD-specific codes. Prior to converging to a final RD phenotypic algorithm, four semiautomated approaches were evaluated. These iterations are described in the Supplementary Materials and in (Supplementary Fig. 1).

**Fig. 2:**
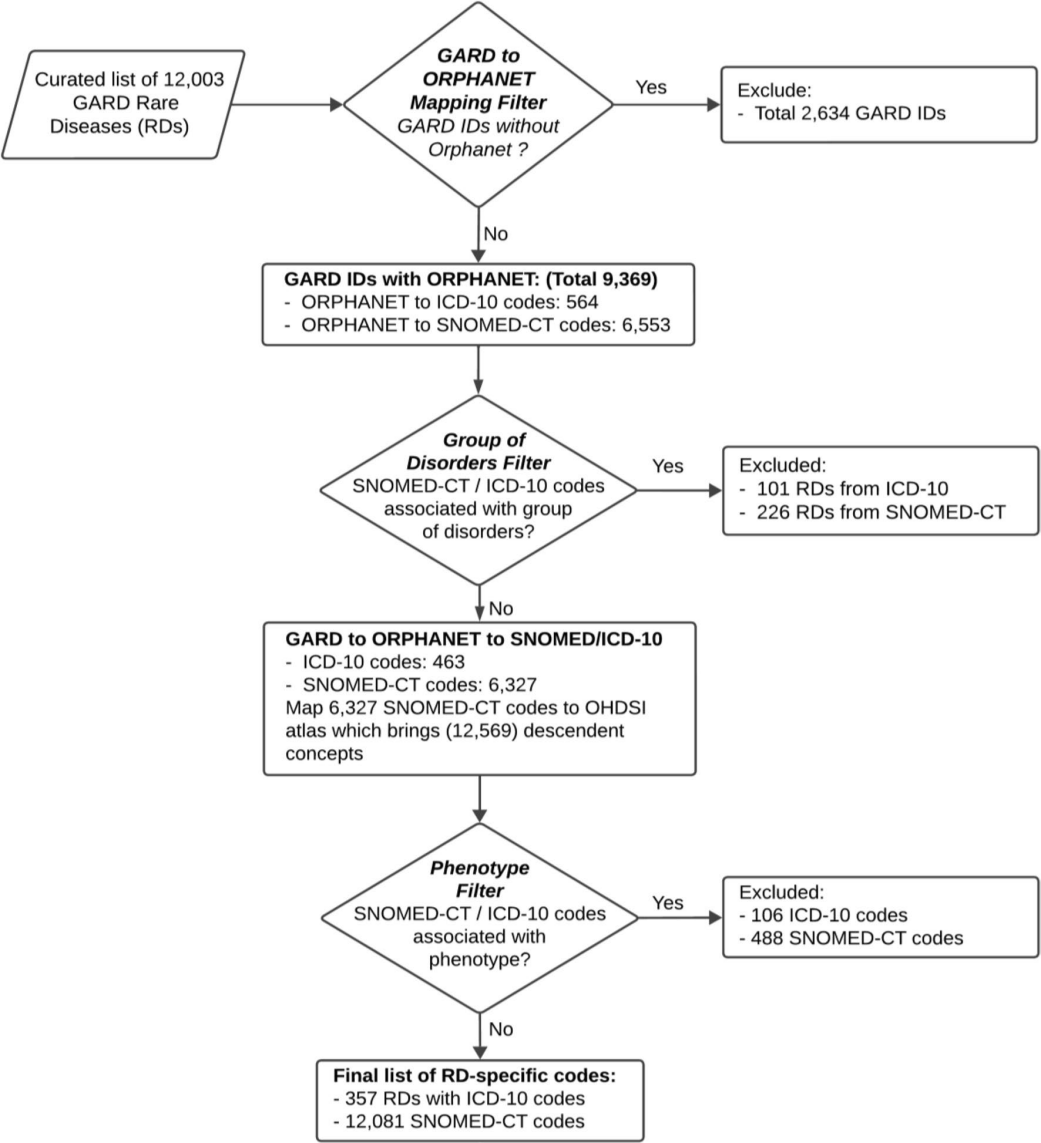
RD phenotyping Algorithm. A semi-automated algorithm was built to define RD-specific ICD-10 and SNOMED-CT codes. The algorithm starts with 12,003 RDs, each defined by a unique GARD ID, which are mapped to ORPHANET to retrieve associated ICD-10 and SNOMED-CT codes. At this step, groups of disorders and broad concepts (e.g., one-to-many mappings) are removed. Next, a phenotype exclusion step is performed after pulling in descendent concepts of the remaining SNOMED-CT codes. As a result, 357 ICD-10 codes and 12,081 SNOMED-CT codes are produced, representing at least 6,342 RDs.

In the final algorithm (Figs. 1 and 2), we first removed RDs associated with the tag of “group of disorders” (e.g., Disorder of carbohydrate metabolism has 396 descendent record counts and is thus tagged as “group of disorders”) based on the ORPHANET definition. Second, we expanded the list of codes to include descendent concepts of our remaining 6,327 SNOMED-CT codes by searching for SNOMED-CT codes using the Observational Health Data Sciences and Informatics (OHDSI) Atlas tool^28^. We assumed that parent concepts are RDs and that their corresponding descendent concepts are thus also RDs. We then curated descendent concepts to filter out those concepts that were represented in the Human Phenotype Ontology (HPO)^29^ via the HPO API (accessed February 25^th^, 2025). Filtering out phenotypes ensures that the resulting codes represent the specific disease itself rather than manifestations or characteristics of the disease which could be shared across multiple diseases (e.g., sensory motor neuropathy, muscle weakness, etc.). The application of these final filtering steps resulted in the final list of RD-specific codes that can be utilized to identify RDs in any EHR system. See further details in the Supplementary Materials.

Resulting SNOMED-CT codes from all 4 phenotypic algorithms were evaluated against a manually curated list of 1,715 RD concept IDs. This curated list subset contains the concepts that were mappable in another cohort (in All of Us research program V7) and thus may not fully represent all rare diseases.To create this list, we conducted comprehensive manual reviews by cross-referencing multiple authoritative rare disease knowledge bases to verify disease classification as rare. In addition to the estimated prevalence in the US general population, the prevalence of each disease in large scale population-based cohorts was also considered during this verification process.

### Definitions of RD classes based on ORPHANET linearization

The ORPHANET linearization classification, accessed through Orphadata (July 2023 version), was used to produce groups of RDs with similar etiologies. The RD-specific SNOMED-CT and ICD-10 codes were directly mapped to the ORPHANET linearization. When a disease is present in multiple classes, the linearization rules for ORPHANET classifications prioritize the most severely affected body system, the most determining involvement for prognosis, and finally the specialist most likely to be relied on for the management of the disease^30^. The number of RDs mapped to the linearization is based on the number of unique GARD IDs prior to phenotype filtering.

We also retrieved RD related publications from the Rare Disease Alert System (RDAS) [https://rdas.ncats.nih.gov], an RD based integrative research data platform, to access the research effort made on those RDs. GARD IDs were applied to search for relevant publications via the RDAS API (accessed September 9th, 2024) [https://rdas.ncats.nih.gov/apis/publications].

### Establishing the COVID-19 cohort in the N3C enclave

The National COVID Cohort Collaborative (N3C)^15^ is an aggregated clinical data resource with participating data partners in the U.S., harmonized using the Observational Medical Outcomes Partnership (OMOP) data model, and subjected to quality reviews and checks. All patients within the N3C Enclave possess historical data from the same healthcare system dating back to January 1, 2018. This dataset includes information on preexisting health conditions (e.g., comorbidities) and other relevant medical history (lookback data)^31^. For this study, we used N3C enclave data tables from version V154 which includes 21,704,702 patients. Patients with COVID-19 were identified as those with a positive COVID-19 diagnosis, based on reverse transcription polymerase chain reaction (RT-PCR) or Antigen (Ag) or U07.1 diagnosis tests, between January 1st, 2020, and January 4th, 2024. The following exclusion criteria were then applied to construct the COVID-19 cohort in this study: 1) patients with missing or invalid data on sex (missing) and age (missing or ages outside the range [2–118]) were removed; 2) patients with no encounter visit before and after COVID-19 diagnosis date were removed; and 3) removed records from data partners with data that did not meet N3C quality control criteria (Supplementary Fig. 2). The N3C Data Enclave is approved under the authority of the National Institutes of Health Institutional Review Board. Each N3C site maintains an institutional review board–approved data transfer agreement.

### Identifying RD patients in the COVID-19 cohort

We map our RD-specific SNOMED-CT codes to the OMOP concept table to obtain the corresponding OMOP concept IDs. These were then linked, along with the ICD-10 codes, to the condition occurrence table, allowing us to identify patients diagnosed with a rare disease (RD) prior to their COVID-19 diagnosis within our COVID-19 cohort. An additional RD prevalence filter was applied to remove diseases with a prevalence rate greater than 6/10,000, which corresponds to the definition of RD in the United States of a prevalence < 200,000 individuals nationwide^35,36^, (200,000 / 342,375,461 U.S. population^37^ is approximately 6 per 10,000). Furthermore, patients associated with the ORPHANET linearization class of “rare disease due to toxic effects” were removed, because this linearization contained a mix of etiologies and disease types (e.g., complications from a medical product, environmental exposures, substance use and abuse), rendering interpretation of this group difficult. Patients associated with the “rare transplantation disease” group were removed because the group was limited to diseases that are a comorbidity or complication of having had a transplant (e.g., aneurysm of the vein of the transplanted kidney). Other patients with “rare odontologic disease” and “rare abdominal surgical disease” were removed due to missing mortality information for those patients available in N3C. The “Rare genetic disease” and “rare infertility” classes were not evaluated because no patients mapped to those classes. “Rare surgical cardiac disease” mapped only one SNOMED-CT code which was a phenotype and hence excluded. “Rare surgical thoracic” and “maxillo-facial diseases” were also removed because they had fewer than 20 mortality events, which fell below the N3C threshold for analysis. After exclusion of these classes, 21 linearization classes remained for our use case analysis in N3C.

### Statistical assessments

The severity outcomes evaluated were COVID-19 related mortality and hospitalization, both of which were encoded as binary variables. Mortality was defined using the broader N3C criteria, which includes both in-hospital or hospice deaths and deaths reported through external sources (e.g., government mortality: death certificates and person-reporting, ObituaryData.com, Private Obituary: Obituary data sourced from funeral homes, newspapers, and other online obituary sources sourced from other private sources). Including these sources ensures a more comprehensive mortality capture and aligns with standard practices. Hospitalization was defined as positive for patients with a hospitalization stay within 16 days of the date of COVID-19 diagnosis ^13,16^.

The demographic variables used to adjust model included: 1) age in years categorized as 1–20, 21–40, 41–65, > 65 years and 2) body mass index (BMI, in *kg*/${m}^{2}$) categorized as underweight (< 18.5), normal (18.5–25), overweight (25–30), BMI obese (≥ 30). The baseline characteristic demographic table (Table 1) was created with the R libraries (gtsummary (2.1.0), tidyverse (2.0.0) & gt (0.11.1) libraries)^32^. Logistic regression models were applied to evaluate association between each RD class and COVID-19 outcomes (e.g., mortality, hospitalization). These models were adjusted for age and BMI. The model was specified as:

$$\mathit{log}\left(\frac{P(Y=1)}{1-P(Y=1)}\right)={\beta }_{i,0}+{{\beta }_{i,1}*RDClass}_{i}+\sum_{j=1}^{4}{\alpha }_{i,j}*{X}_{j}+\sum_{k=1}^{4}{\mu }_{i,k}*{X}_{k,i=1,\dots ,21.}$$

where ${RDClass}_{i}$ is a binary variable indicating presence of the ${i}^{th}$ RD class and ${X}_{j}$ denotes covariates for age group and ${X}_{k}$ for BMI. Odds ratios (ORs), 95% confidence intervals (CIs), and p-values were reported for each disease-specific model. Statistical significance was defined as p-values < 0.001 (corresponding to a Bonferroni adjusted p-value of 0.05). Statistical modeling was conducted within the N3C enclave using SQL, Python (3.10.16), statsmodels (0.14.4), Patsy (1.0.1), Numpy (1.26.4), Pandas (1.5.3) and Spark SQL (3.4.1.34).

**Table 1:** Characteristics of Coronavirus Disease 2019 positive patients with and without preexisting diagnosis of a rare disease.

Characteristic/Demographic	Overall (N = 4,835,718)	Rare disease (No) (N = 4,518,882)	Rare disease (Yes) (N = 316,836)	OR (95% CI)	*p* value*
Age, n (%)					
Age (2–20)	756,843 (15.65%)	720,618 (15.95%)	36,225 (11.43%)	0.68 (0.673, 0.688)	0.00E + 00
Age (20–40)	1,298,094 (26.84%)	1,230,274 (27.23%)	67,820 (21.41%)	0.728 (0.722, 0.734)	0.00E + 00
Age (40–65)	1,765,726 (36.51%)	1,649,547 (36.50%)	116,179 (36.67%)	1.007 (1.0, 1.015)	6.24E-02
Age (> 65)	1,015,055 (20.99%)	918,443 (20.32%)	96,612 (30.49%)	1.72 (1.706, 1.733)	0.00E + 00
BMI, n (%)					
BMI underweight (< 18.5)	175,712 (3.63%)	162,234 (3.59%)	13,478 (4.25%)	1.193 (1.172, 1.215)	5.66E-83
BMI normal (18.5—25)	549,293 (11.36%)	507,462 (11.23%)	41,831 (13.20%)	1.202 (1.19, 1.215)	7.47E-251
BMI overweight (25—30)	775,317 (16.03%)	714,899 (15.82%)	60,418 (19.07%)	1.254 (1.242, 1.265)	0.00E + 00
BMI obese (≥ 30)	3,335,396 (68.97%)	3,134,287 (69.36%)	201,109 (63.47%)	0.768 (0.762, 0.773)	0.00E + 00
Sex, n (%)					
Female	2,903,745 (60.05%)	2,704,480 (59.85%)	199,265 (62.89%)	1.137 (1.129, 1.146)	1.33E-250
Male	1,931,973 (39.95%)	1,814,402 (40.15%)	117,571 (37.11%)	0.879 (0.873, 0.886)	1.33E-250
Race, n (%)					
Asian	169,248 (3.50%)	159,932 (3.54%)	9,316 (2.94%)	0.826 (0.808, 0.843)	2.63E-70
Black or African American	638,220 (13.20%)	584,613 (12.94%)	53,607 (16.92%)	1.371 (1.357, 1.384)	0.00E + 00
Missing/Unknown/Other	573,602 (11.86%)	540,117 (11.95%)	33,485 (10.57%)	0.871 (0.86, 0.881)	6.16E-120
White	3,454,648 (71.44%)	3,234,220 (71.57%)	220,428 (69.57%)	0.908 (0.901, 0.915)	3.52E-128
Ethnicity, n (%)					
Hispanic or Latino	573,977 (11.87%)	543,263 (12.02%)	30,714 (9.69%)	0.786 (0.776, 0.795)	0.00E + 00
Not hispanic or Latino	3,905,198 (80.76%)	3,640,151 (80.55%)	265,047 (83.65%)	1.235 (1.224, 1.247)	0.00E + 00
Missing/Unknown	356,543 (7.37%)	335,468 (7.42%)	21,075 (6.65%)	0.917 (0.904, 0.93)	9.26E-33
Smoking status, n (%)					
Current or former smoker	819,634 (16.95%)	767,140 (16.98%)	52,494 (16.57%)	0.971 (0.962, 0.981)	3.29E-09
Non-smoker	4,016,084 (83.05%)	3,751,742 (83.02%)	264,342 (83.43%)	1.03 (1.02, 1.04)	3.29E-09
Death, n (%)	105,920 (2.19%)	88,667 (1.96%)	17,253 (5.45%)	2.877 (2.83, 2.926)	0.00E + 00
Hospitalized, n (%)	255,429 (5.28%)	221,449 (4.90%)	33,980 (10.72%)	2.331 (2.303, 2.36)	0.00E + 00

* Pearson’s Chi-squared test.

## Results

Our main goal was to build a semi-automated phenotyping algorithm that produces RD-specific ICD-10 and SNOMED-CT codes (Fig. 1). These codes are key to identifying RD patients in any EHR system that leverages them, thereby enabling analyses of numerous RDs together (See Supplementary materials on how to apply our phenotyping algorithm in local OMOP database). We exemplify this utility in a COVID-19 case study where we conducted a clinical assessment of RD patients in the N3C Enclave, including COVID-19 related mortality and hospitalization (Fig. 1).

### Phenotyping algorithm for the systematic identification of RD patients in EHR systems

As a starting point, a list of RDs, based on the US definition, was derived by mapping a curated list of 12,003 GARD IDs to ORPHANET, resulting in 9,369 RDs with 6,553 SNOMED-CT codes and 564 ICD-10 codes. Subsequently, four algorithms were tested (Supplementary Fig. 1), and for each, various combinations of filters were applied to remove phenotypes, groups of diseases and common diseases. Our goal was to produce a final algorithm that reduced the reliance on manual curation while retaining as many RDs as possible. Supplementary Fig. 3 shows the number of ICD-10 and SNOMED-CT codes that are returned in common and different between each approach. The drop in the number of codes returned by our final approach is due to group of disorders and phenotypes.

We further compared the resulting SNOMED-CT codes from our semiautomated approaches to those obtained from a manually curated list of 1,715 diseases (Supplementary Table 1). Importantly, our final approach showed the largest percent of diseases mappable to this curated subset list at 89.8%. Of these, 11.6% were determined not RD (false positives), leaving the large majority, 88.4% as true RDs. While other approaches greatly minimize this false positive rate (as low as 1.0%), this approach comes at the cost of low mapping rate (as low as 42.6% compared to 89.8% for our final approach). We conclude that overall, our final algorithm (Fig. 2) minimized the amount of manual curation, number of phenotypes and number of groups of diseases, and number of false positives resulting in an optimized approach to producing RD-specific codes for the identification of RD in EHRs.

We applied our final algorithm to the initial 6,553 SNOMED-CT and 564 ICD-10 codes. This resulted in 101 ICD-10 and 226 SNOMED-CT codes removed because they are associated with a group of disorders, and 106 ICD-10 and 488 SNOMED-CT codes were removed because they are associated with phenotypes (as per available HPO mapping) rather than diseases. In total, a final list of RD-specific codes comprised 357 ICD-10 codes and 12,081 SNOMED-CT IDs, representing a total of 6,342 unique RDs. This list is available in Supplementary Table 2, along with associated GARD IDs, names and ORPHANET linearization classes and further details on each filtering step are found in the Supplementary Materials.

### Characteristics of diseases represented by RD-specific SNOMED/ICD-10 codes

**Fig. 3:**
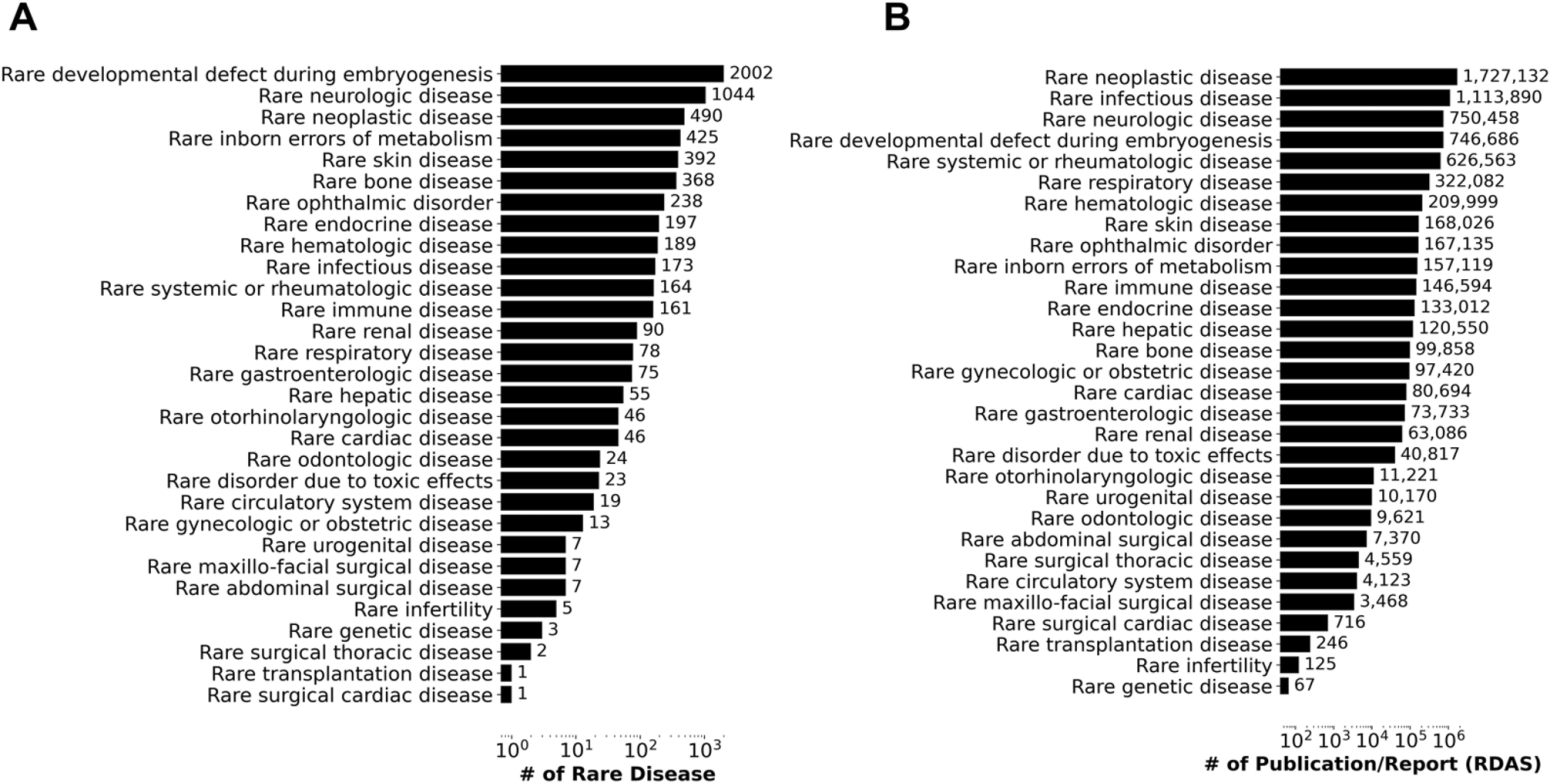
Characteristics of the 6342 unique RDs represented by RD-specific ICD-10 and SNOMED-CT codes. (**A**) Number of diseases by ORPHANET linearization; (**B**) Number of publications/reports from (RDAS).

At least 6,342 diseases (estimated prior to the phenotype filter) with RD-specific codes were categorized via ORPHANET linearization (Supplementary Table 3). Because each ORPHANET RD maps to a single ORPHANET linearization, these categories are useful for evaluating groups of diseases and for clinical assessment. (Fig. 3A) depicts the number of RDs per category for the 30 linearization classes that represent the 6,342 diseases. Seven linearization classes are poorly represented (See Methods).

The most well represented classes of RD are “rare developmental defects during embryogenesis” and “rare neurologic disease" with 2002 and 1,044 RDs in each, respectively. The least represented linearization includes rare urogenital, rare maxillo-facial surgical and rare surgical thoracic diseases, with fewer than 10 ICD-10 and SNOMED-CT codes. The remaining linearization classes include 13 to 490 unique codes.

The number of representative codes for each linearization class is shown in Supplementary Table 4. The following eight classes are represented by < 10 SNOMED-CT and ICD-10 codes: rare abdominal surgical disease, rare maxillo-facial surgical disease, rare urogenital disease, rare infertility, rare genetic disease, rare surgical thoracic disease, rare surgical cardiac disease, rare transplantation disease. Interestingly, the number of RD-specific codes for each linearization aligns well with how well these diseases are evaluated in the literature. Figure 3B shows the number of associated reports/publications obtained via ORPHANET linearization. The most widely reported (with > 750,000 publications/reports) categories include rare neoplastic, rare infectious and rare neurological diseases, which are also the top 3 represented linearization by our resulting RD-specific codes. The 3 least represented linearization classes in the literature, with fewer than 250 publications, are rare transplantation, infertility and rare genetic disease.

### Use case: evaluation of RD patients in a COVID-19 cohort using the N3C enclave

We exemplify the utility of our list of RD-specific codes by studying a large population of patients with RDs in the N3C enclave which comprises 21,704,702 patients, with 8,463,370 having a positive COVID-19 diagnosis (Supplementary Fig. 2, Methods). After applying exclusion criteria based on missing data on age and sex, < 1 encounter visits before/after COVID-19 diagnosis date, and data that did not pass N3C quality control, our final COVID-19 cohort comprised 4,835,718 patients. As a final step, we further applied filtering criteria based on prevalence, ensuring that only diseases with a prevalence rate less than 6/10,000 were represented (See Methods). In total, 8 ICD-10 codes and 19 SNOMED-CT codes were removed (Supplementary Fig. 4).

We next cross-linked our list of RD-specific codes resulting from our phenotypic algorithm to stratify patients into those with or without a preexisting RD diagnosis. Of the RD-specific codes (12,081 SNOMED-CT and 357 ICD-10 codes) identified by our algorithm (Supplementary Figure S4), 10,592 SNOMED-CT and 349 ICD-10 codes were mappable to N3C and passed the prevalence filter. In total, we identified 316,836 patients (6.55%) with a preexisting RD, while 4,518,882 did not have a preexisting RD (Supplementary Fig. 2, Table 1). Given the heterogeneity of RDs, we further stratified patients into the 21 ORPHANET linearization (Supplementary Fig. 5A). The rare neoplastic disease group shows the largest representation in N3C (N = 79,021), followed by rare systemic or rheumatologic disease (N = 47,375) and rare hematologic disease (N = 37,579). Conversely, rare circulatory system disease (727), and rare urogenital diseases (N = 693) were the least represented. The number of patients in the remaining groups ranged from 3,226 to 32,606. Notably, no patients mapped to the ORPHANET linearization classes of rare genetic disease and rare infertility, both of which are represented by < 10 codes.

Globally, among the 316,836 patients with preexisting RD, 33,980 (10.72%) were hospitalized and 17,253 (5.45%) died. These patients with preexisting RD were more likely to be female than male (62.89% female in the RD group vs 59.85% female in the non-RD group), and their age distributions differed (for example, 11.43% under 20 years in the RD group vs 15.95% under 20 years in the non-RD group) from those of patients without preexisting RD. The demographic data are shown in Table 1, which reveals significant differences in age, BMI, race, ethnicity, and smoking status between patients with and without preexisting RD. Furthermore, higher rates of mortality (5.45% vs 1.96% in non-preexisting RD patients) and hospitalization (10.72% vs 4.90% in non-preexisting RD patients) were observed in patients with preexisting RD (Table1). The distribution of mortality for patients with and without preexisting RDs was also evaluated by age and sex categories to reveal potential differences between those two demographics (Supplementary Fig. 6).

We next evaluated the impact of age and sex on mortality of patients with and without preexisting RDs. For all age groups, we observed a higher mortality between patients with and without RD in both males and females, although the increasing rate of mortality is more pronounced in males than in females (Supplementary Fig. 6). For example, in the 65 + age group, the percent mortality difference between patients with and without preexisting RD is higher in males by 4.22% (Chi-square p-value < 0.0001) and higher in females by 2.2% (Chi-square p-value < 0.0001). We also observe an increase in the percent mortality difference as patients get older in all patients, although the increase is more pronounced for patients with preexisting RD. For example, male patients with preexisting RD show a 10.69% increase in mortality between those aged 1–20 years those aged 65 + years (Chi-square p-value < 0.0001) while patients without preexisting RD Similarly, female patients with preexisting RD showed a 7.35% increase between the 1–20 years and 65 + age groups while those without preexisting RD showed an increase of 6.1% between the same age groups (Chi-square p-value < 0.0001). Similar observations are made when evaluating the hospitalization rate.

The rates of mortality and hospitalization were then explored for 21 classes except for those classes excluded because of insufficient mortality data or a lack of patient mapping (see Methods). Our analysis revealed the highest mortality rates among patients with rare neoplastic diseases (10.4%) and rare respiratory diseases (9.0%). The highest hospitalization rates were observed in patients with rare endocrine disorders (15.4%) and rare respiratory diseases (15.0%). (Supplementary Fig. 5B and C). The least affected linearization classes for both mortality and hospitalization rates (calculated as the number of mortalities/hospitalizations within a linearization class divided by the total number of mortalities/hospitalizations across all the linearization classes in our N3C cohort) include rare gynecologic or obstetric diseases (0.5% mortality and 6.2% hospitalization). The next least percentages were observed for rare otorhinolaryngologic diseases at 2.1% mortality and rare skin disease at 7.9% hospitalization.

Multivariable logistic regression models, adjusted for age and BMI, were applied to assess whether RD classes showed increased or decreased risk of mortality and hospitalization **(**Table2). For mortality, the highest odds were observed for rare cardiac diseases (OR = 4.07, 95% CI: 3.48–4.76), rare otorhinolaryngologic diseases (OR = 4.00, 95% CI: 3.24–4.92), and rare respiratory diseases (OR = 3.92, 95% CI: 3.76–4.09). All other RD classes demonstrated significantly elevated mortality risk (all *p* < 0.0001) except rare gynecologic or obstetric and rare urogenital.

**Table 2:** Multivariate logistic regression model of COVID-19 Severity Outcomes (Mortality/ Hospitalization), adjusted for age and BMI.

	Mortality			Hospitalization	
Rare disease class	(95% CI)	*p* value	Rare disease class	(95% CI)	*p* value
Rare cardiac diseases	4.07 (3.48–4.76)	< 0.0001	Rare otorhinolaryngologic disease	4.31 (3.89–4.77)	< 0.0001
Rare otorhinolaryngologic disease	4.00 (3.24–4.92)	< 0.0001	Rare endocrine disease	3.38 (3.10–3.70)	< 0.0001
Rare respiratory disease	3.92 (3.76–4.09)	< 0.0001	Rare circulatory system disease	3.21 (2.58–4.00)	< 0.0001
Rare endocrine disease	3.34 (2.93–3.81)	< 0.0001	Rare respiratory disease	3.08 (2.98–3.19)	< 0.0001
Rare bone diseases	2.92 (2.56–3.34)	< 0.0001	Rare developmental defect during embryogenesis	3.06 (2.93–3.19)	< 0.0001
Rare neoplastic disease	2.90 (2.80–3.01)	< 0.0001	Rare hematologic disease	2.93 (2.84–3.02)	< 0.0001
Rare developmental defect during embryogenesis	2.71 (2.52–2.91)	< 0.0001	Rare cardiac diseases	2.67 (2.40–2.98)	< 0.0001
Rare hematologic disease	2.64 (2.52–2.76)	< 0.0001	Rare bone diseases	2.46 (2.23–2.72)	< 0.0001
Rare circulatory system disease	2.64 (1.83–3.79)	< 0.0001	Rare renal disease	2.30 (2.05–2.57)	< 0.0001
Rare infectious disease	2.56 (2.40–2.72)	< 0.0001	Rare gynecologic or obstetric disease	2.17 (1.98–2.37)	< 0.0001
Rare hepatic disease	2.43 (2.22–2.65)	< 0.0001	Rare infectious disease	2.16 (2.06–2.26)	< 0.0001
Rare gastroenterologic disease	2.35 (2.16–2.56)	< 0.0001	Rare gastroenterologic disease	2.10 (1.97–2.25)	< 0.0001
Rare neurologic disease	2.03 (1.96–2.10)	< 0.0001	Rare neurologic disease	1.98 (1.93–2.02)	< 0.0001
Rare systemic or rheumatologic disease	1.94 (1.86–2.02)	< 0.0001	Rare hepatic disease	1.90 (1.77–2.03)	< 0.0001
Rare immune disease	1.82 (1.65–2.00)	< 0.0001	Rare ophthalmic disorder	1.88 (1.76–2.00)	< 0.0001
Rare renal disease	1.82 (1.52–2.18)	< 0.0001	Rare systemic or rheumatologic disease	1.85 (1.79–1.90)	< 0.0001
Rare inborn errors of metabolism	1.77 (1.52–2.06)	< 0.0001	Rare inborn errors of metabolism	1.82 (1.66–2.00)	< 0.0001
Rare gynecologic or obstetric disease	1.63 (1.19–2.25)	0.0024	Rare neoplastic disease	1.81 (1.75–1.87)	< 0.0001
Rare ophthalmic disorder	1.50 (1.37–1.65)	< 0.0001	Rare urogenital disease	1.73 (1.34–2.24)	< 0.0001
Rare skin disease	1.30 (1.20–1.42)	< 0.0001	Rare immune disease	1.64 (1.53–1.77)	< 0.0001
Rare urogenital disease	1.18 (0.76–1.84)	0.4535	Rare skin disease	1.20 (1.13–1.28)	< 0.0001

All RD classes showed increased risk of hospitalization with a range of odds ratios of 1.20 to 4.31 (Table2). The strongest associations were observed for rare otorhinolaryngologic diseases (OR = 4.31, 95% CI: 3.89–4.77), rare endocrine diseases (OR = 3.38, 95% CI: 3.10–3.70), and rare circulatory system diseases (OR = 3.21, 95% CI: 2.58–4.00).

## Discussion

Although numerous research articles have been published on identifying RD patients in EHR systems, there remains a notable scarcity of automated approaches capable of identifying a wide array of RDs on a large scale. To address this gap, we have developed an algorithm to automate the identification process of RDs in extensive EHR systems. The major outcome of this study is thus the development of a phenotyping algorithm that produces RD-specific codes, specifically 12,081 SNOMED-CT codes and 357 ICD-10 codes representing at least 6,342 RDs. To the best of our knowledge, this is the largest number of RDs represented by SNOMED-CT and ICD-10 codes. These RDs represent 30 ORPHANET classes, although 8 classes are under-represented with < 10 codes. Availability of these codes allows investigation of RDs as groups, rather than individuals or small numbers of RDs, in any EHR system that include SNOMED-CT and ICD-10. This capacity empowers clinicians and researchers to find novel shared treatment options for this difficult to study population.

Our phenotype algorithm addresses major challenges in systematically defining RDs in EHR systems. First, we eliminated RD codes that relate to group of disorders that could erroneously bring in common diseases when we expanded the code list with their descendent, and we also removed those that relate to phenotypes. Second, we focused on minimizing manual curation so that the process can be readily executed when updates to source databases (e.g., GARD, ORPHANET, OHDSI) become available. Third, recognizing the lack of convergence in the definition of RDs across the world, we anchored our algorithm to GARD IDs and thus follow the US definition of RDs. While our algorithm produces a comprehensive list of RD codes, we acknowledge that some RDs might be missed due to exclusion criteria applied. In addition, as we minimize manual curation, we recommend careful evaluation of codes when used to create a cohort. In our case study evaluation COVID-19 outcomes in N3C, we added a prevalence filter to remove potentially erroneously represented RDs in the EHR system. This filter removed 26 codes (Supplementary Table 5) which represent common diseases. In the end, the prevalence of RD patients in our cohort is 6.55%, which is in line with the US prevalence rate of 6%^1^.

A recent study by Thygesen et al^17^ evaluated prevalence, clinical, and demographic data for RD using a large retrospective observational study in England. An algorithm specific to their study was designed to identify RD using ORPHANET and implement filters to remove certain disease types (e.g., clinical group, clinical subtype, etiological subtype, and biological anomaly, etc.) and mappings without existing codes in the observational study. While some key similarities exist between the algorithm presented in Thygesen et al. and ours (e.g., use of ORPHANET, prevalence and removal or groups/broad disorders), our algorithm produces codes that are independent of a specific cohort. Therefore, our list of RD-specific codes can be used directly as a starting point for analyses in any EHR system.

We note that there is a large skew in the distribution of the numbers of RD-specific codes and associated publications by linearization classes (Fig. 3, Supplementary Table 4). Surprisingly, while 80% of RDs are related to genetic disorders, only 3 RD-specific codes map to that category^33^. Notably, this underrepresentation is an artifact of the rare genetic disease linearization, and the priority rules applied when mapping a disease to a linearization^30^. The ORPHANET linearization categories are useful for grouping together diseases that undergo similar clinical care or organs affected. In fact, if an RD corresponds to multiple categories, priority is given to organ-based or clinical system classifications over etiology (e.g., genetic basis)^30^. For this reason, the genetic RD classification only has several codes mapping to it. If this linearization were to be further analyzed with a need to include all genetic RD, the grouping of this genetic RD category could be revised by incorporating cross-ontology mappings in the MONDO ontology. Other underrepresented codes linearization classes could also be affected by these linearization class rules.

As a use case of this list of RD-specific codes, we identified RD patients in a COVID-19 cohort established within the N3C enclave to broadly compare the demographics of COVID-19 patients with and without preexisting RDs and to evaluate the odds ratio of COVID-19 related hospitalization and mortality in patients with and without preexisting RDs. Our analysis, while preliminary and not adjusted for variables such as vaccination status or antiviral treatment, demonstrates the practical utility of these codes in evaluating COVID-19 severity across RD categories. To the best of our knowledge, this preliminary work represents the largest study in the United States that evaluates COVID-19 severity outcomes in relation to RD classes at scale. Multivariable logistic regression adjusted for age and BMI revealed that several RD classes, most notably rare cardiac, and otorhinolaryngologic diseases, were associated with substantially higher odds of both hospitalization and mortality. These findings align with prior work by Thygesen et al., who also observed elevated mortality risk among patients with rare developmental, renal, neurologic, immune, ophthalmic, systemic/rheumatologic, skin, and bone diseases (their models were adjusted for age and sex). Our results also align with a comprehensive analysis in Hong Kong showing that RD patients had an increased risk of COVID-19 related mortality compared with the general population (models were adjusted for age) ^34^. Notably, stratified analyses within the Hong Kong cohort indicated that mortality risk was particularly elevated among RD patients aged ≥ 60 years, aligning with our observation of increased mortality in RD patients aged > 65 years.

Several limitations of this work regarding the phenotyping algorithm and use case are worth noting. Our approach relies on ORPHANET classification and further filtering of ICD-10 and SNOMED-CT codes specific to RDs. We recognize that removing codes that map to phenotypes (e.g., HPO terms) could remove valid RDs. The phenotype filter was applied to avoid inflating RD prevalence with non-specific symptoms (e.g., “muscle weakness” in muscular dystrophy). Thus, we prioritized specificity over sensitivity, thereby enhancing diagnostic precision. We also note that our performance calculations (88.4% true RD codes) are based on a subset of 1,715 RDs which may not be representative of all RDs. The performance for all individual codes is not known. In future work, we aim to refine this phenotyping strategy by incorporating contextual co-occurrence rules (e.g., symptom + disease codes) and leveraging natural language processing (NLP) methods to better balance sensitivity and specificity in RD phenotyping. Limitations of our case study include the lack of adjustment for vaccination status, exposure to antivirals, and other relevant clinical factors and these limitations will be addressed in future studies. Further, the N3C data are aggregated from multiple healthcare systems, which use four common data models with varying levels of granularity. Harmonizing these disparate data requires making assumptions and inferences, which could introduce systematic biases. We also recognize that RD patients are underrepresented in EHR systems due to heterogeneity of the coding systems used in different clinical settings. Patients with suspected RDs or undiagnosed conditions cannot be identified with these codes. Pediatric rare diseases are outside the scope of the current N3C use case and that findings should not be extrapolated to very young pediatric populations. Finally, the OMOP Common Data Model (CDM) leveraged here in our algorithm does not capture all EHR data elements, which may lead to incomplete representation of certain clinical variables, thereby affecting both disease classification and outcome ascertainment. Collectively, the use of an RD subset for evaluating performance of our algorithm and the potential underrepresentation of RD subgroups in the data impacts the generalizability of our findings. Validation for specific diseases may thus be required.

Overall, the focus of this work was to develop and implement a phenotyping algorithm to produce RD-specific codes and to apply these codes to demonstrate their utility in identifying RD patients in large clinical cohorts. Results from our approach provide a robust foundation for broader evaluation of RDs in EHR systems that utilize commonly used codes. Evaluating groups of RDs are advantageous to help increase statistical power for finding clinically relevant patterns and to better understand RDs as a complex system rather than single diseases. Our use case evaluation in COVID-19 provided preliminary findings that further highlight the need for tailored monitoring of RD patients to prevent worse COVID-19 outcomes. Moving forward, this framework provides a crucial foundation for further investigations to replicate findings and to carefully evaluate comorbidities and other COVID-19 relevant variables and outcomes (e.g., vaccination status, long COVID, etc.).

## Supplementary Information

Supplementary Information 1.

Supplementary Information 2.

## Data Availability

Data used in this manuscript are available through the N3C Enclave as described in section N3C Acknowledgements. See https://github.com/arjunyadaw/Rare-Disease-Cohort-Building-in-EHR-System.git for the code repository.
