# Supplementary material for: Systematic identification of rare disease patients in electronic health records enables evaluation of clinical outcomes
Source: Sci Rep. 2026 Apr 18;16:18987. doi: 10.1038/s41598-026-43020-x (PMC13276054; doi:10.1038/s41598-026-43020-x)
Supplement: Supplementary file 2 — Supplementary Information 2. [file 41598_2026_43020_MOESM2_ESM.docx]

**Systematic identification of rare disease patients in electronic health records enables evaluation of clinical outcomes**

Arjun S. Yadaw^1^, Eric Sid^2^, Hythem Sidky^1^, Chenjie Zeng^3^, Qian Zhu^1^, Ewy A. Mathé^1^, On behalf the N3C Consortium

^1^National Center for Advancing Translational Sciences (NCATS), NIH, Rockville, MD, USA

^2^Division of Rare Diseases Research Innovation, National Center for Advancing Translational

Sciences (NCATS), National Institutes of Health, Bethesda, MD 20892, USA.

^3^Precision Health Informatics Section, National Human Genome Research Institute, National Institutes of Health, Bethesda, MD, 20814

**Supplementary Methods**

Unique rare diseases (RDs) were defined by 12,003 GARD IDs, which follow the definition of a prevalence rate fewer than 6/10,000 in the United States^35,36,37^. Of the 12,003 RDs, 9,369 mapped to ORPHANET (July 2023 version) and had associated SNOMED-CT and ICD-10 codes. The 2,634 remaining RDs with GARD IDs that did not map to ORPHANET were excluded.

The SNOMED-CT and ICD-10 codes associated with the 9,369 GARD IDs represent single diseases only. As such, descendent RDs are not represented, and additional cross-linking and filtering is necessary to capture RD subtypes that would otherwise be missed. We tried various approaches to 1) remove group of disorders through ORPHANET annotations; 2) maximize the number of RDs represented by including descendent concepts through SNOMED-CT; 3) eliminate phenotypes by matching through the Human Phenotype Ontology (HPO); 4) reduce the amount of manual curation necessary; 5) remove reliance on cohort-specific prevalence calculations which could be prone to biases of patient and disease representation. Supplementary Figure 1 describes the various approaches tried, leading to the final approach which meets our goals.

To apply the phenotyping algorithm in a local OMOP database, users can follow:

- Direct Use of Codes: Researchers can directly use the *SNOMED-CT* codes listed in *Supplementary Table S2* and the *ICD-10* codes in *Supplementary Table S3* to build RD cohorts within their local EHR or OMOP database.
- Custom Rare Disease Lists: If a researcher wishes to use their own list of rare diseases, our phenotyping algorithm can be applied from scratch by following the workflow outlined in Figure 2 and Supplementary Figure 4.
- Implementation Details: Detailed instructions and code examples for running the algorithm are provided in our [GitHub repository]:

( <https://github.com/arjunyadaw/Rare-Disease-Cohort-Building-in-EHR-System.git>).

**
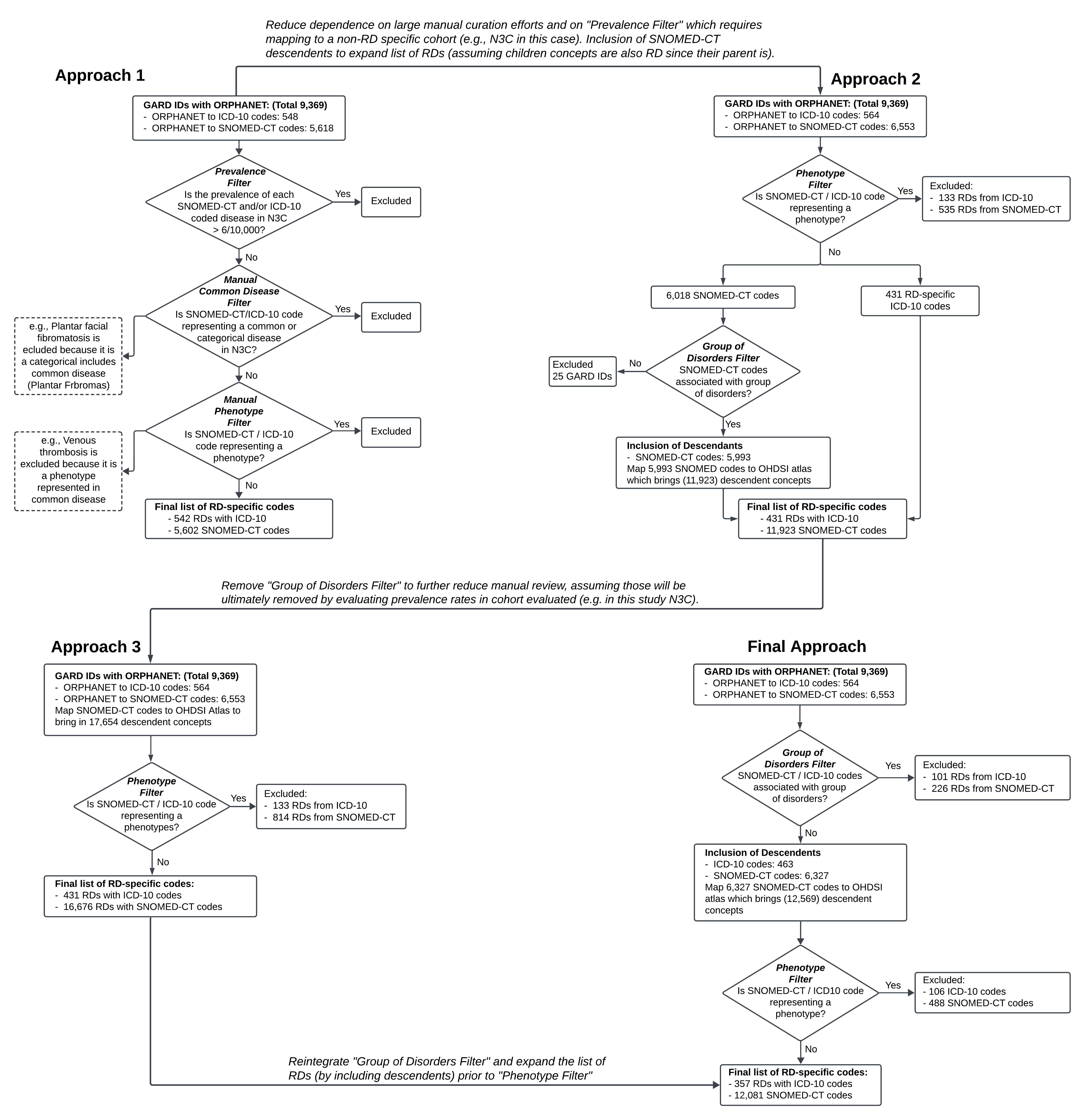
**

**Supplementary Figure 1: Evolution of semi-automated approaches to produce RD-specific SNOMED-CT and ICD-10 codes.** In Approach 1, we mapped the starting ICD-10 and SNOMED-CT codes (representing 9,369 IDs) to N3C to remove RDs with an prevalence > 6/10,000 (following the US definition of RDs). Subsequently, we manually curated the remaining RDs and removed common diseases and phenotypes. This approach was not ideal because it required large curation efforts and relied on prevalence rates of a specific cohort (in this case N3C). Further, we were missing RDs because the initial codes oftentimes did not include RD descendent concepts (which can be extracted from SNOMED-CT). In Approach 2, we started by removing phenotypes, then curated the resulting list manually to remove groups of disorders. In the final step, we brought in descendent concepts. This approach still required substantial manual curation so in Approach 3, we removed the manual curation to remove groups of disorders as we assumed that those would be excluded through the prevalence filter that can be applied when mapping codes to EHR. This approach introduced too many false positives so in the final approach, we removed groups of disorders programmatically, where groups of disorders are defined as having more than one descendent (noting that this is prior to the expansion of descendent codes through the OHDSI Atlas). After this filter, the inclusion of descendent codes and exclusion of phenotypes was performed. This final approach also removed the reliance of a prevalence filter based on a specific cohort (in this case N3C), which could introduce false positives or negatives due to RD representation biases.


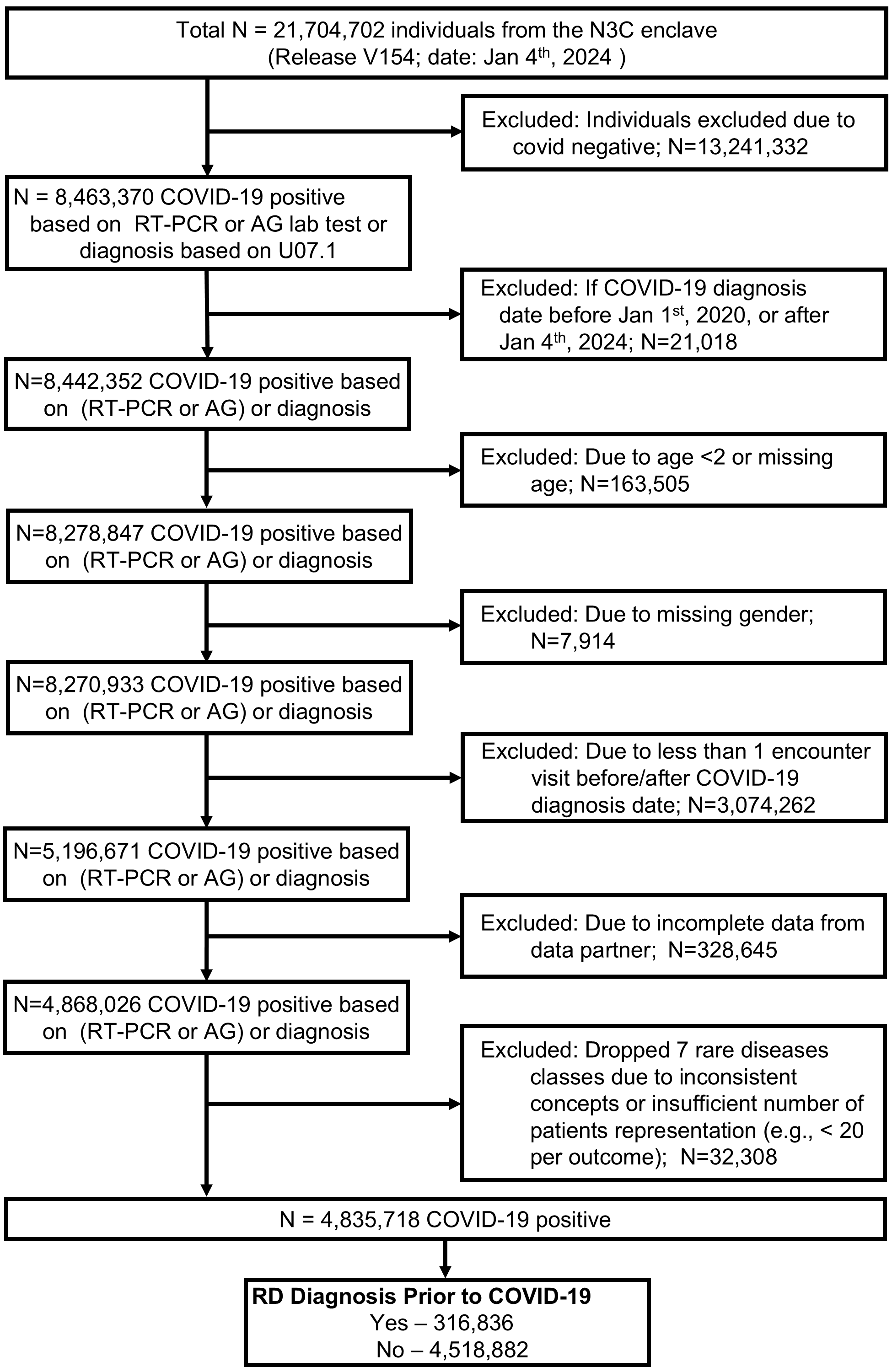


**Supplementary Figure 2: Workflow to define COVID-19 patients with and without RD in N3C.** Of the 21,704,702 patients in the N3C enclave released version V154, 4,835,718 were defined as COVID-19 positive between January 1^st^, 2020, to January 4^th^, 2024, as confirmed by a RT-PCR or Antigen test or diagnosis test. This subset also passed the following filters: non missing values for age and sex, at least one encounter visits before or after covid diagnosis date, incomplete data from data partners. Lastly, patients with COVID-19 were stratified into two groups, those with and without RDs, based on our generated SNOMED-CT and ICD-10 codes.


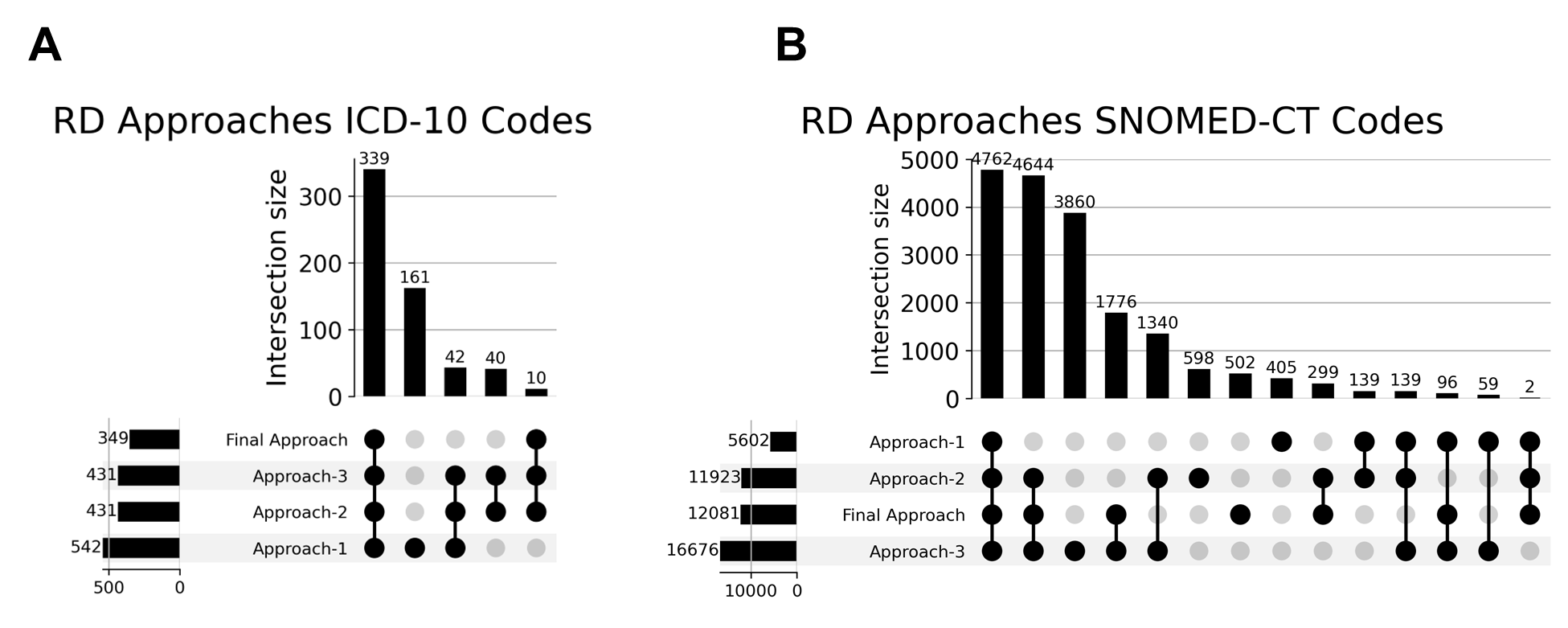


**Supplementary Figure 3: UpSet plots showing common and unique ICD-10 (A) and SNOMED-CT codes (B) across the 4 approaches tested (shown in Supplementary Figure 1).** A total of 243 ICD-10 and 6,531 SNOMED-CT codes resulting from approaches 1, 2, and/or 3 were filtered out in the final approach.

**
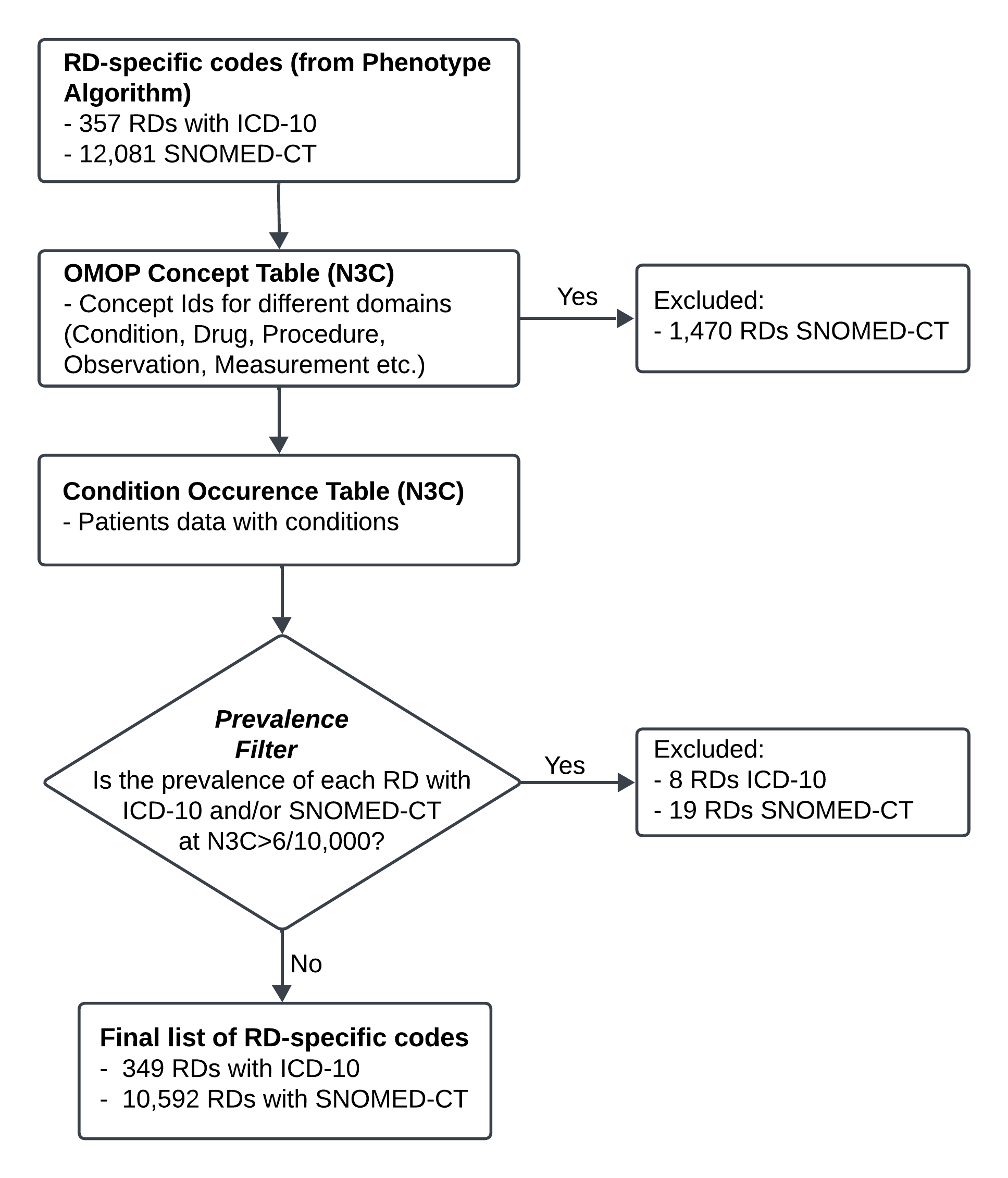
**

**Supplementary Figure 4:** **Workflow describing the use of RD-specific codes to define RD patients in N3C.**

**
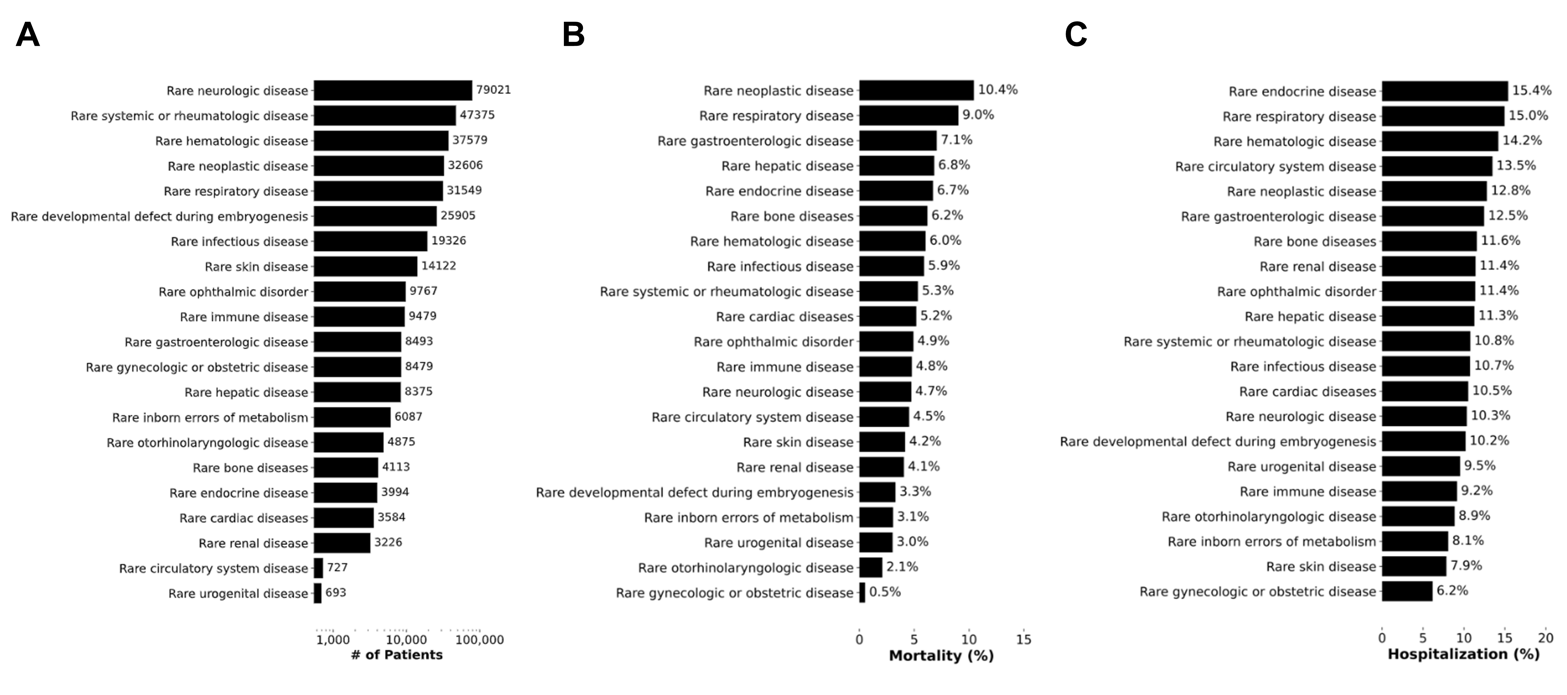
Supplementary Figure 5: Description of patients with preexisting RD identified in the N3C COVID-19 cohort.** A) Number of patients per RD ORPHANET linearization class for RD patients identified within our N3C COVID-19 cohort. B) and C) Distribution of the number of RD patients with a COVID-19 diagnosis stratified by COVID-19 related mortality (B) and hospitalization (C). The mortality/hospitalizations rates were calculated as the number of mortalities/hospitalizations within a linearization class divided by the total number of mortalities/hospitalizations across all the linearization classes in our N3C cohort.


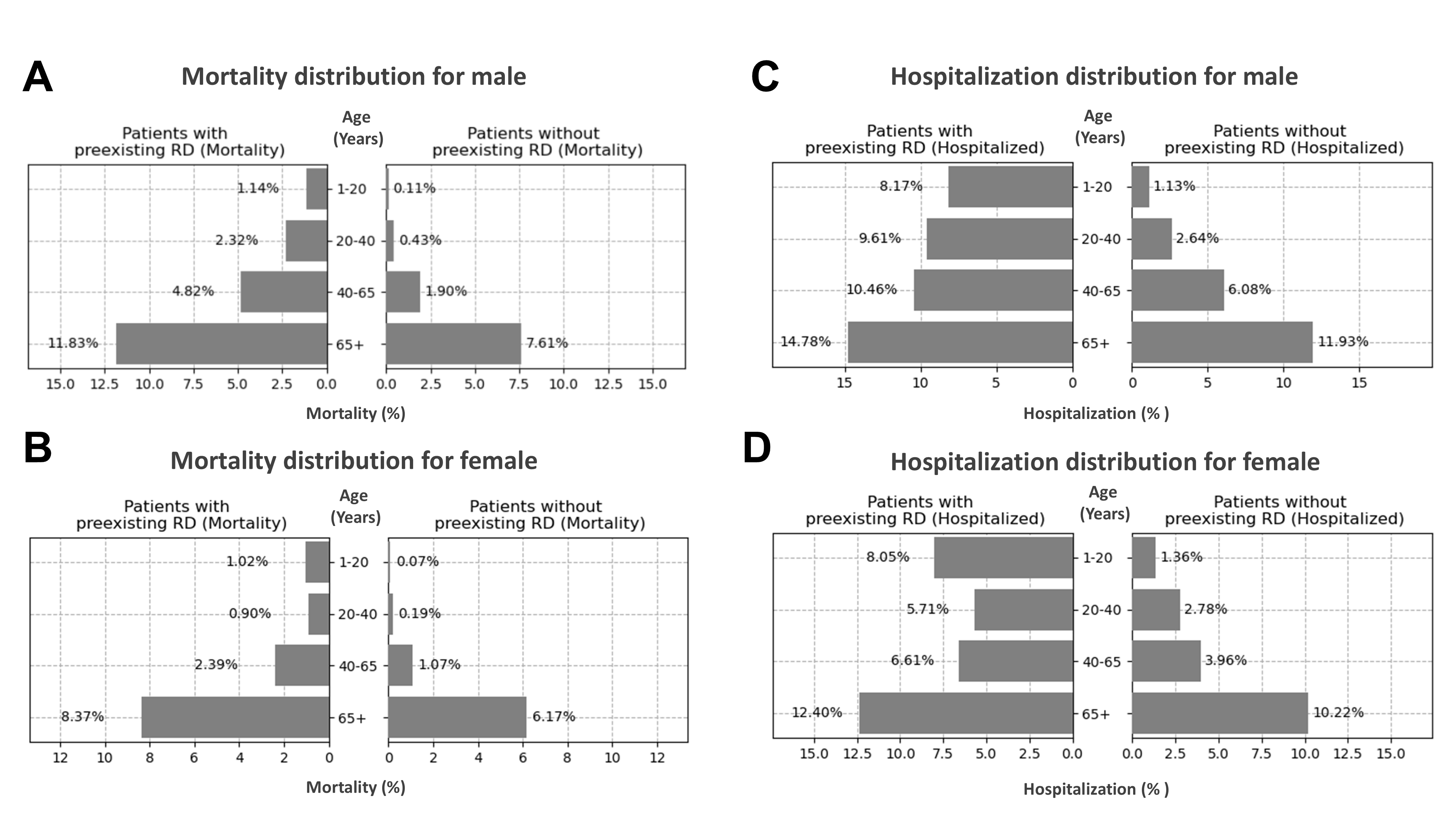


**Supplementary Figure 6: Distribution of the number of patients with preexisting RD that have suffered COVID-19 related death or hospitalization, stratified by age and sex.** Distribution of percent mortality of COVID-19 patients with/without preexisting RD in males (A) and females (B), stratified by age groups. Distribution of percent hospitalized COVID-19 patients with/without preexisting RD in males (C) and females (D), stratified by age groups.
